# Internet Addiction among Students: cross-sectional study

**DOI:** 10.1192/j.eurpsy.2025.975

**Published:** 2025-08-26

**Authors:** Z. Nesrine, I. Gassara, R. Feki, N. Smaoui, N. Charfi, J. Ben Thabet, L. Zouari, M. Maalej, S. Omri, M. Maalej

**Affiliations:** 1 pédopsychiatrie; 2 psychiatrie C, hopital hedi chaker, Sfax, Tunisia

## Abstract

**Introduction:**

Conceptually, the internet has transformed the Earth into a vast information network village, significantly enhancing human experience through unprecedented availability and exchange of information. However, the potential adverse effects of internet addiction on human health have emerged as a major global concern.

**Objectives:**

This study aimed to estimate the prevalence of internet addiction among students.

**Methods:**

A cross-sectional, descriptive, and analytical study was conducted between October 2022 and January 2023 among students from various faculties in Sfax. Data were collected through a self-administered electronic questionnaire accessible online, created using the Google Forms application. The questionnaire explored sociodemographic and relational data. Internet addiction was assessed using the Internet Addiction Scale (IAS).

**Results:**

The average age of the students was 25,62 ± 3,29 years, with a sex ratio of 1/5. Among the participants, 96% resided in urban areas, and 81,9% lived with their families. Nearly half of the students were from the Sfax Faculty of Medicine, and 64,4% were in the third cycle of their studies. The study found a mean total score of 74,27 +/- 21,25 on the IAS, indicating an estimated prevalence of internet addiction at 24,2%. Factors correlated with internet addiction included excessive internet use by family members (p=0,004) and poor adaptation to the faculty (p=0,03).

**Conclusions:**

Internet addiction was prevalent in our student population. Exploring the characteristics associated with this addiction would undoubtedly assist in identifying the risks our students might face.

**Disclosure of Interest:**

None Declared

